# Characterization and In Vivo Antiangiogenic Activity Evaluation of Morin-Based Cyclodextrin Inclusion Complexes

**DOI:** 10.3390/pharmaceutics15092209

**Published:** 2023-08-26

**Authors:** Federica De Gaetano, Fatima Margani, Vincenzina Barbera, Valeria D’Angelo, Maria Paola Germanò, Venerando Pistarà, Cinzia Anna Ventura

**Affiliations:** 1 Dipartimento di Scienze Chimiche, Biologiche, Farmaceutiche e Ambientali, Università di Messina, Viale Ferdinando Stagno d’Alcontres 31, I-98166 Messina, Italy; fedegaetano@unime.it (F.D.G.); valeria.dangelo@unime.it (V.D.); mariapaola.germano@unime.it (M.P.G.); 2 Dipartimento di Chimica, Materiali e Ingegneria Chimica “G. Natta”, Politecnico di Milano, Via Mancinelli 7, I-20131 Milano, Italy; fatima.margani@polimi.it (F.M.); vincenzina.barbera@polimi.it (V.B.); 3 Dipartimento di Scienze del Farmaco e della Salute, Università di Catania, Viale A. Doria 6, I-95125 Catania, Italy

**Keywords:** morin, cyclodextrins, inclusion complexes, antiangiogenic activity, in vivo studies, chick chorioallantoic membrane, zebrafish embryos

## Abstract

Morin (MRN) is a natural compound with antiangiogenic, antioxidant, anti-inflammatory, and anticancer activity. However, it shows a very low water solubility (28 μg/mL) that reduces its oral absorption, making bioavailability low and unpredictable. To improve MRN solubility and positively affect its biological activity, particularly its antiangiogenic activity, in this work, we prepared the inclusion complexes of MNR with sulfobutylether-*β*-cyclodextrin (SBE-*β*-CD) and hydroxypropyl-*β*-cyclodextrin (HP-*β*-CD). The inclusion complexes obtained by the freeze-drying method were extensively characterized in solution (phase-solubility studies, UV–Vis titration, and NMR spectroscopy) and in the solid state (TGA, DSC, and WAXD analysis). The complexation significantly increased the water solubility by about 100 times for MRN/HP-*β*-CD and 115 times for MRN/SBE-*β*-CD. Furthermore, quantitative dissolution of the complexes was observed within 60 min, whilst 1% of the free drug dissolved in the same experimental time. ^1^H NMR and UV–Vis titration studies demonstrated both CDs well include the benzoyl moiety of the drug. Additionally, SBE-*β*-CD could interact with the cinnamoyl moiety of MRN too. The complexes are stable in solution, showing a high value of association constant, that is, 3380 M^−1^ for MRN/HP-*β*-CD and 2870 M^−1^ for MRN/SBE-*β*-CD. In vivo biological studies on chick embryo chorioallantoic membrane (CAM) and zebrafish embryo models demonstrated the high biocompatibility of the inclusion complexes and the effective increase in antiangiogenic activity of complexed MRN with respect to the free drug.

## 1. Introduction

The most important goal of pharmaceutical research is to obtain new therapies and reduce the spread of the disease and associated mortality. In recent years, great attention has been paid to natural molecules because they have extraordinary properties and unique benefits [1,2,3]. They are safe, have few adverse effects [4], and have special health benefits [5]. Many natural compounds have attracted attention for their anticancer effect and very low side effects, largely demonstrated by in vitro and in vivo studies [6]. Therefore, their combination with chemotherapy drugs can represent an effective antitumor strategy, inhibiting tumor growth and multidrug resistance and achieving synergistic pharmacological action [7]. Natural molecules are also used to design totally natural drug delivery systems, such as unsaturated fatty acid vesicles exploiting their potential as lipophilic drug carriers [8].

Flavonoids are natural substances with unique tricyclic structures, divided into several categories based on their structure. Their bioavailability and biological activity are influenced by the presence of hydroxyl groups; in fact, through resonance, these groups can donate electrons, stabilize free radicals and mediate the antioxidant effect [9]. Flavonoids have demonstrated potential effects in neurodegeneration and neuroinflammation [10]; cancer treatment and immunomodulation [11,12]; arrhythmias [13]; obesity [14]; biofilm treatment [15]; bacterial [16], fungal [17], and viral infections [18]; inflammation [19]; angiogenic process [20]; and many others.

**Figure 1:**
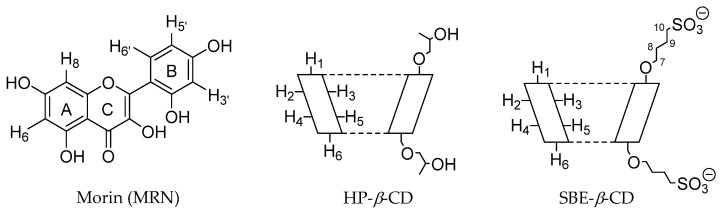
Molecular structure of MRN and schematic structure of HP-*β*-CD and SBE-*β*-CD.

Morin (MRN; 3,5,7,7,2′,4′-pentahydroxyflavone) (Figure 1) is a flavonoid with various protective systemic effects, i.e., neuroprotective, cardioprotective, nephroprotective, and others [21]. It has significant anticancer properties too [22], showing low toxicity even in the case of chronic treatment [21]. Moreover, MRN exhibits significant biofilm inhibition and anti-quorum sensing activities against resistant bacterial strains [23,24]. In multidrug therapy, MRN reduces the adverse effects of co-administered synthetic drugs without altering their function [21]. MRN modulates many cytokines and several cellular signaling pathways, including the inhibition of the release of inflammatory cytokines IL-6, IL-8, of the tumor necrosis factor (TNF) and nuclear factor kappa-light-chain-enhancer of activated B cells (NF-κB) [25]. Jung et al. [26] demonstrated that MRN displayed significant inhibition of chick chorioallantoic membrane (CAM) angiogenesis, showing similar activity to retinoic acid used as a positive control. These extraordinary properties suggest that MRN can be used in the treatment and prevention of many human pathologies. Unfortunately, it is very soluble in methanol (50 mg/mL) but poorly soluble in water (28 μg/mL) [27]. This unfavorable physical–chemical property reduces the oral bioavailability of the drug and prevents the realization of liquid formulations for oral or parenteral administration, consequently limiting its clinical applications [28].

To increase the water solubility and bioavailability of natural substances, different supramolecular systems could be used, such as solid lipid nanoparticles [29,30], unsaturated fatty acid vesicles [8], polymeric nanoparticles [31,32], liposomes [33], and cyclodextrins (CDs) [34]. Among these, CDs, alone or in combination with other supramolecular nanostructures, play a principal role in pharmaceutical technology to improve the solubility in biological fluids and the bioavailability of synthetic drugs and natural active molecules [35,36,37,38,39]. Kazlauskaite et al. [40] demonstrated *α*-, *β*- and *γ*-CDs can be used as an eco-friendly method for isoflavones solubilization in plant extracts. They significantly improve the extraction of daidzein and genistein from *Trifolium pratense* L. Other authors [41] described the ability of CDs to include natural antioxidants, protecting their oxidation by pro-oxidant agents, thus improving their ability to prevent the enzymatic browning of food. Moreover, it seems that CDs can act as “secondary antioxidants”, reducing food browning and enhancing the naturally occurring antioxidant capacity of the food itself [41].

Recently, the preparation and characterization of inclusion complexes of MRN with native *β*-CD, 2-hydroxypropyl-*β*-CD (HP-*β*-CD), and heptakis(2,6-O-dimethyl)-*β*-CD (DM-*β*-CD) were described by Jullian et al. [42], demonstrating a different interaction of MRN with the native and modified CDs. Subsequently, Lima et al. [43] reported improved in vivo antihyperalgesic and anti-inflammatory effects of MRN because of its complexation with HP-*β*-CD.

MRN also shows significant antiangiogenic activity [26], playing a potential role in all angiogenesis-dependent diseases, such as cancer disease. Thus, we remained very interested in assaying the effect of the complexation into CDs on the water solubility and in vivo antiangiogenic activity of MRN. We selected two modified CDs, sulfobutylether-*β*-CD (SBE-*β*-CD) and HP-*β*-CD (Figure 1), approved by the FDA for parenteral administration, with the aim of preparing liquid formulations for oral or parenteral administration. To the best of our knowledge, there are no studies in the literature concerning the complexation of MRN with SBE-*β*-CD, and no paper has been published concerning the antiangiogenic activity of MRN complexed with any CDs, neither in vitro nor in vivo. Despite a physical–chemical characterization of the MRN/HP-*β*-CD inclusion complex reported by other authors [42,43], before proceeding with the biological in vivo assay, a characterization of our prepared MRN/HP-*β*-CD system is needed to confirm the existence of an inclusion complex. Therefore, both inclusion complexes were prepared by freeze-drying method and deeply characterized in solution (phase-solubility studies, UV–Vis titration, and NMR spectroscopy) and in the solid state (differential scanning calorimetry and X-ray diffraction). The biocompatibility and antiangiogenic activity of complexed MRN were evaluated by in vivo studies on chick embryo chorioallantoic membrane (CAM) and zebrafish embryo models.

## 2. Materials and Methods

### 2.1. Materials

Morin (MRN, C_15_H_10_O_7_, molecular weight, 302.24 g/mol) and 2-hydroxypropyl-*β*-cyclodextrin (HP-*β*-CD, 0.6 molar substitution, average molecular weight, 1380), retinoic acid, 2-methoxyestradiol (ME), endogenous alkaline phosphatase (EAP), tris buffer (pH 7.4) and dimethyl sulfoxide (DMSO) were purchased from Sigma-Aldrich (St. Louis, MO, USA). Sulfobutylether-*β*-cyclodextrin (SBE-*β*-CD, CAPTISOL^®^, the average degree of sulfobutyl substitution is seven, average molecular weight, 2162) was kindly supplied by CyDex Pharmaceutical (Lenexa, KS, USA). Water used throughout this study was double distilled, then filtered through 0.22 μm Millipore^®^ GSWP filters (Bedford, MA, USA). All other products and reagents were of analytical grade.

### 2.2. Preparation of the Inclusion Complexes

The MRN/HP-*β*-CD and MRN/SBE-*β*-CD inclusion complexes were prepared by the freeze-drying method, starting with a hydro-alcoholic solution containing the drug and CDs in a molar ratio 1:3. Briefly, HP-*β*-CD (136.62 mg, 1 M^−4^) and SBE-*β*-CD (214 mg, 1 M^−4^) were solubilized separately, at room temperature, in 8 mL of water. MRN (10 mg, 0.33 M^−4^) was solubilized at room temperature, in the dark, in 2 mL of methanol, and dropped to CD solutions under magnetic stirring. The solutions were divided into 10 vials and lyophilized for 72 h (VirtTis Benchtop K Instrument, SP Scientific, Gardiner, NY, USA).

### 2.3. Phase-Solubility Studies

Ten milliliters of aqueous solution, containing increasing amounts of HP-*β*-CD or SBE-*β*-CD (0 to 0.012 M), were added of solid MRN in amount exciding its intrinsic solubility (28 μg/mL). The suspensions were placed in a thermostatic bath (Telesystem 15.40, Thermo Scientific, Waltham, MA, USA), equipped with a temperature control unit (Telemodul 40C, Thermo Scientific, Waltham, MA, USA), at 25.0 ± 0.5 °C, in the dark, and under magnetic stirring, for 48 h, until the equilibrium was reached. After that, the suspensions were filtered through Sartorius Minisart-SRP 15-PTFE filters, 0.22 µm (Bedford, MA, USA), and the solutions were analyzed by UV–Vis spectroscopy for MRN quantification (see Section 2.4). The experiments were conducted in triplicate. No degradation of MRN was observed during the experimental time. The concentration of MRN detected in the solutions was plotted against the CD concentration, and phase-solubility diagrams were constructed. Apparent 1:l association constants (*K_c_*) of both complexes were calculated according to the Higuchi and Connors equation (Equation (1)), where S_0_ is the intrinsic water solubility of MRN [44]:

(1) $$K_{c}=\frac{Slope}{\left(1-Slope\right)S_{0}}$$

### 2.4. Quantification of MRN

The amount of MRN in the solution was determined by UV–Vis spectroscopy by using a StellarNet BLACK-Comet Model C diode array spectrophotometer (StellarNet, Inc., Tampa, FL, USA), employing one-centimeter rectangular quartz cells (Hellma, Milan, Italy). UV–Vis spectra were performed in the spectral range 200–600 nm in methanol/water 20/80 (*v*/*v*), and the calibration curve was constructed with concentration ranging from 0.0008 mg/mL to 0.05 mg/mL (R^2^ equal to 0.9980).

### 2.5. Determination of Dissolution Rate

In accordance with the 44th United States Pharmacopoeia (USP) paddle method, dissolution studies were conducted at 37 ± 0.5 °C, under magnetic stirring (100 rpm). Briefly, an amount of free MRN (100 mg) or a corresponding amount in the complexes was suspended in 900 mL of water. At predetermined times (15, 30, 45, 60, and 120 min), aliquots of the medium were collected, filtered (Sartorius Minisart-SRP 15-PTFE, 0.22 µm filters, Bedford, MA, USA), and the concentration of MRN in solution was determined by UV–Vis spectroscopy. In order to maintain the sink conditions, the volume was adjusted to 900 mL with fresh preheated medium. The experimental data were reported as an average of at least three experiments.

### 2.6. UV–Vis Titration

Free MRN (0.066 × 10^−3^ M) and increasing concentrations of HP-*β*-CD or SBE-*β*-CD (0.066, 0.198, 0.33, 0.66, 1.32, 3.3 and 6.6 × 10^−3^ M), were solubilized in a methanol/water mixture 20/80 (*v*/*v*) and stirred in the dark at 500 rpm before analysis for 24 h at 25.0 ± 0.5 °C. The solutions were analyzed by UV–Vis spectroscopy in the spectral range 200–600 nm.

### 2.7. NMR Spectroscopy

Samples of equivalent concentrations (8 mM) of MRN, SBE-*β*-CyD, HP-*β*-CD, and the corresponding 1:1 inclusion complexes were prepared in a D_2_O/CD_3_OD (1:1, *v*/*v*) solution and transferred to 5 mm NMR tubes for spectrum acquisition. All spectra were recorded at 300 K with a Varian Unity Inova 500 MHz (11.75 T) instrument. The deuterated methanol (3.30 ppm) was used as internal reference, to avoid the addition of external ones that could interact with the CDs.

### 2.8. Dynamic Light Scattering (DLS)

Determination of the sizes of the CDs and the inclusion complexes nanoaggregates in water were performed by using a Malvern Zetasizer Nano ZS instrument with a He–Ne laser of 4 mW power and 633 nm wavelength and an avalanche photodiode detector. Triplicate measurements were carried out for all samples, each averaging at least 10 runs at 25 °C. HP-*β*-CD, SBE-*β*-CD, and their inclusion complexes with MRN were analyzed at a concentration ranging from 0.01 to 1 mg/mL. The volume size distribution was utilized for aggregate state analysis.

### 2.9. Zeta Potential (ζ)

The zeta potential (ζ) values were measured by using a Zetasizer Nano ZS (Malvern Instrument, Malvern, UK) with a 633 nm red laser and a power of 5.0 mW. Triplicate measurements were carried out.

### 2.10. Wide-Angle X-ray Diffraction (WAXD)

An automatic Bruker D8 Advance diffractometer, with nickel-filtered Cu–Kα radiation, was used to record WAXD patterns, obtained in reflection, in 4–90° as the 2θ range, with 2θ being the peak diffraction angle.

### 2.11. Thermogravimetric Analysis (TGA)

Thermal properties were studied using a Perkin Elmer STA 6000 instrument. Analyses were performed according to the standard method ISO9924-1. Samples (5–10 mg) were heated under nitrogen (30 mL min^−1^) from 30 °C to 300 °C, at a heating rate of 10 °C min^−1^, kept at 300 °C for 10 min, and then heated up to 550 °C at 20 °C min^−1^. After being maintained at 550 °C for 15 min, they were further heated up to 900 °C with a heating rate of 10 °C/min and kept at 900 °C for 3 min, then kept at 900 °C for 30 min under flowing air (60 mL/min).

### 2.12. Fourier-Transform Infrared (FT-IR)

The FT-IR Nicolet iS5 spectrometer (Thermo Scientific, Madison, WI, USA) was used to collect FT-IR spectra with a wavenumber ranging from 4000 cm^−1^ to 400 cm^−1^. To record the spectra at different temperatures, the samples were analyzed using the same spectrometer equipped with an attenuated total reflection accessory (Pike Technologies, Madison, WI, USA) on a heatable germanium crystal (up to 120 °C) in transmission mode (64 scans and 4 cm^−1^ resolution). The chosen temperature range was 25–100 °C, with a heating and cooling rate of 10 °C/min.

### 2.13. In Vivo Studies

#### 2.13.1. Chick Chorioallantoic Membrane (CAM) Assay

CAM assay was performed following the method of Certo et al. [45]. Fertilized chicken eggs were incubated at 37.0 ± 0.5 °C for 4 days. After incubation, a window (1 cm^2^) was carefully created on the broad side of each egg to check by a visual inspection of the chorioallantoic membrane. For the assay, stock solutions of MRN in DMSO were prepared and diluted to 33 μM using Tris buffer (pH 7.4). The final concentration of DMSO was maintained at 0.2% (*v*/*v*). Complexed MRN in HP-*β*-CD and SBE-*β*-CD was solubilized at the same concentration of free in Tris buffer (pH 7.4). All samples were applied directly to the CAM surface at a volume of 100 µL/egg). The control group received only DMSO (0.2%, *v*/*v*). Retinoic acid (3 µM) was used as a positive control. Ten eggs were used for each group. After 48 hours of exposure at 37.0 ± 0.5 °C, the microvasculature of each egg was observed under a stereomicroscope (SMZ-171 Series, Motic, Hong Kong, China). The images were acquired by a digital camera (Moticam^®^ 5 plus). After counting the number of blood vessel branch points in a standardized area, the antiangiogenic activity was calculated using the equation 1-T/C, where T represents the number of vessel branch points in the treated CAMs, whereas C indicates the number of vessel branch points in control samples. The results obtained were finally expressed as percent values vs. negative control. Each experiment was repeated three times.

#### 2.13.2. Zebrafish Embryo Culture and Treatment Protocol

Adult male and female zebrafish were maintained in flowthrough aquaria at 28.5 °C ± 0.5 °C, on a 14/10 h (light/dark) photoperiod, according to the standard protocol [46]. Three times a day, they were fed with live brine shrimp (*Artemia salina*). The eggs generated by natural mating were collected and examined under a microscope. The fertilized eggs containing embryos with normal morphology were selected and used in subsequent experiments. They were manually dechorionated with forceps and distributed in 96-well microplates (one embryo per well) containing 200 µl embryo medium. From 24 to 72 h post fertilization (hpf), the embryos were exposed to the following treatments: free MRN or complexed with HP-*β*-CD and SBE-*β*-CD at concentrations ranging from 50 to 150 µM. 2-methoxyestradiol (ME, 20 µM) was employed as a standard antiangiogenic substance. MRN and ME were dissolved in DMSO and diluted at fixed concentrations by embryo medium. The final concentration of DMSO was maintained at 0.2% (*v*/*v*) in all samples. The control group received only DMSO (0.2%, *v*/*v*). The inclusion complexes were dissolved in embryo medium. All experiments were performed in compliance with the European Directive 2010/63/EU and the ethical guidelines described in the “National Institutes of Health Guide for Care and Use of Laboratory Animals”.

#### 2.13.3. Quantitative Determination of Endogenous Alkaline Phosphatase (EAP) Activity

Quantitative determination of EAP activity was performed following the method described by Iannuzzi et al. [47]. The embryos at 72 hpf were, firstly, dehydrated with increasing concentrations of ethanol, then they were washed three times with a diethanolamine buffer (1 M, pH 9.8) and incubated with p-nitrophenyl phosphate disodium salt (0.5 mg/mL) for 30 min at room temperature. The reaction was stopped by the addition of NaOH (2 M). The optical density of the soluble end product was measured at 405 nm using a microplate reader (Mutiskan GO, Thermo Scientific). Vessel growth was determined as a percentage in optical density compared with control (Equation (2)) and finally expressed as inhibition %. Each assay was repeated at least three times.

(2) $$\%\;vessel\;formation=\frac{OD\;treated-OD\;control}{OD\;control-OD\;control}\times 100$$

### 2.14. Statistical Analysis

All values are expressed as mean ± standard deviation (SD), and each analysis was performed three times. The results were analyzed by one- and two-way analysis of variance (ANOVA) followed by a Bonferroni post hoc test for multiple comparisons. A value of *p* < 0.05 was considered significant.

## 3. Results and Discussion

CDs are able to increase the solubility of poorly soluble drugs, increasing their bioavailability and pharmacological effect. Thus, they can result in great interest in improving the antiangiogenic activity of the insoluble natural agent, MRN, permitting its clinical employment in angiogenic-dependent diseases, such as tumor growth.

The inclusion complexes of MRN with SBE-*β*-CD and HP-*β*-CD were prepared by freeze-drying a hydro-alcoholic solution containing drug:CD in a 1:3 molar ratio. Methanol was added to permit the solubilization of MRN and to favor the complexation. The high ratio of CDs was chosen to maintain MRN in the solution. The freeze-drying method produced slightly yellow-colored powders that were characterized in an aqueous solution and in the solid state to confirm the complexation.

### 3.1. In Solution Studies

**Figure 2:**
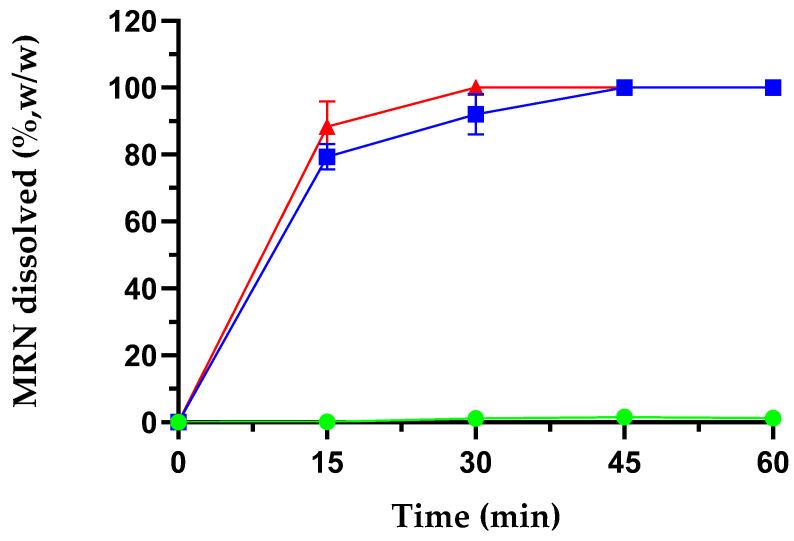
Dissolution profiles of free MRN (green line), MRN/HP-*β*-CD inclusion complex (blue line), and MRN/SBE-*β*-CD inclusion complex (red line) in water at 37.0 ± 0.5 °C. All data related to MRN-inclusion complexes are statistically significant with respect to free MRN data (*p* < 0.001). The results are presented as the mean of three different experiments ± standard deviation (SD). The error bar, if not shown, was within the symbol.

The inclusion complexes showed a significant increase in MRN water solubility of about 100 times for MRN/HP-*β*-CD and 115 times for MRN/SBE-*β*-CD inclusion complexes. The significant improvement in MRN water solubility observed as a result of complexation produced a quantitative dissolution of both complexes within 60 min, whilst about 1% dissolution of free MRN was observed in the same experimental time (Figure 2).

**Figure 3:**
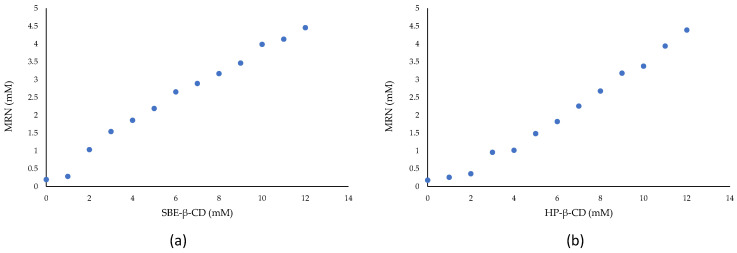
Phase-solubility diagrams of MRN/SBE-*β*-CD (**a**) and MRN/HP-*β*-CD (**b**) complexes in aqueous solution at 25.0 ± 0.1 °C.

Phase-solubility diagrams of MRN in the presence of increasing concentrations of HP-*β*-CD and SBE-*β*-CD are shown in Figure 3. A_L_-type diagrams were obtained for both systems, evidencing the high solubility of the complexes in the range of CD concentrations used (0–12 mM). The slope of both diagrams showed values less than one, evidencing the presence in the solution of the inclusion complexes in a 1:1 molar ratio. By applying the Higuchi and Connors equation [44], the association constants (*Kc*) were determined, obtaining values of 2870 M^−1^ and 3380 M^−1^ for MRN/SBE-*β*-CD and MRN/HP-*β*-CD inclusion complex, respectively.

**Figure 4:**
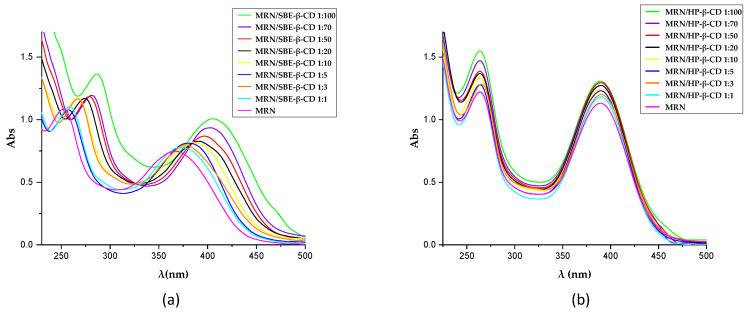
UV–Vis spectra of MRN alone and in the presence of an increasing amount of SBE-*β*-CD (**a**) and HP-*β*-CD (**b**) in a methanol/water mixture 80:20 (*v*/*v*).

The *host*–*guest* interaction between MRN and SBE-*β*-CD and HP-*β*-CD was investigated by UV–Vis spectroscopy. MRN has two bands, the first one at 263 nm (Band II) and the second one at 363 nm (Band I) (Figure 4), attributed to the π→π* transitions of the conjugated benzoyl moiety (rings A–C) and the conjugated cinnamoyl system (rings B–C), respectively [27]. The chemical structure of MRN, with a double bond in the proximity of the carbonyl group in ring C, presents a cross-conjugated system. The delocalization involves separately C and A or C and B rings but not the rings A and B [48]. Different studies demonstrated that the complexation of MRN with proteins [49] or metals [50] results in significant variation in the UV–Vis spectrum of the drug, producing the hyperchromic effect of the bands. Furthermore, the MRN molecule is not planar due to the single bond between the B ring and C ring, which can change its conformation based on the change in its environment [51]. This variation can be followed by a bathochromic effect of the bands of the MRN spectrum [27].

The two different CDs differently influenced the UV–Vis spectrum of MRN, demonstrating a strong *host*–*guest* interaction in both cases. In the presence of increasing concentration of SBE-*β*-CD, we observed a progressive bathochromic and hyperchromic effect on both bands (Figure 4a). The inclusion of MRN into the CD cavity produces a variation in the local polarity of the microenvironment of the chromophore groups of MRN molecule, significatively influencing the delocalization of π electrons with a consequent influence on the intensity and position of the bands. Furthermore, as a consequence of the complexation, the breakdown of the intramolecular and intermolecular hydrogen bonds present in the MRN molecule and the MRN lattice [48,51] and the formation of new hydrogen bonds between cinnamoyl moiety of MRN and the sulfobutyl moiety of the macrocycle could not be excluded. In this way, a conformational change in the C–C bond between the ring B and C could be produced, with substantial modification of this band [27].

The spectra obtained for the MRN/HP-*β*-CD complex clearly demonstrated a different *host*–*guest* interaction with respect to the MRN/SBE-*β*-CD complex. We observed increased intensity of the two MRN bands but no shift of position (Figure 4b). Furthermore, the influence of HP-*β*-CD is more evident in band II rather than band I, showing a greater progressive hyperchromic effect with the increase in the CD concentration. We can hypothesize that HP-*β*-CD well-interacts with the planar portion of the molecule (rings A–C), with little influence on the conformation of the C–C bond between rings B and C.

**Figure 5:**
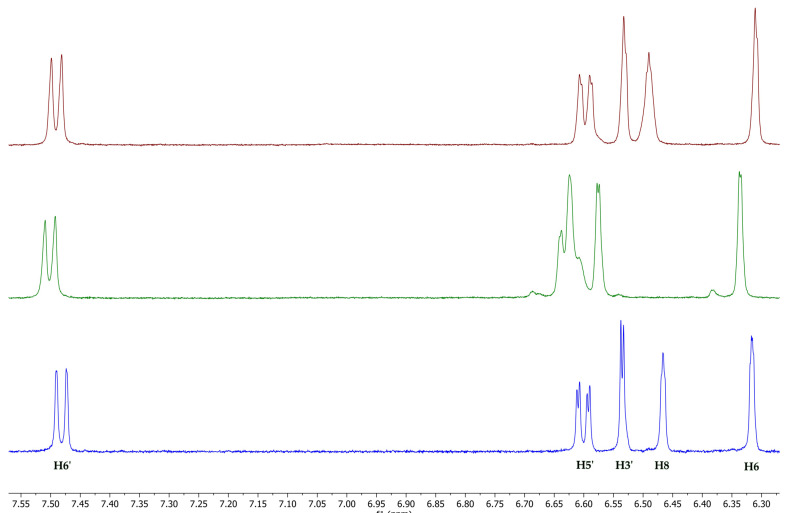
Stacked portions of the ^1^H NMR spectra relative to the free MRN (blue line bottom), MRN/SBE-*β*-CyD (green line), and MRN/HP-*β*-CyD (red line) inclusion complexes. Only those diagnostic signals relative to MRN are shown.

Among the spectroscopic techniques, nuclear magnetic resonance (NMR) is very important to investigate the formation and geometry of the inclusion complexes. During the complexation, the chemical and electronic environments of the protons are modified by the interactions between the host and the guest molecules; therefore, a chemically induced shift of the corresponding protons has been observed. As with most of the substituted CDs, the HP-*β*-CD and SBE-*β*-CD, unfortunately, are a statistical mixture of the different stereoisomers, with unresolved broad peaks, making it almost impossible to follow the chemical shifts of its protons, especially H_3_ and H_5_ protons facing the inside of the cavity, although these were identified through 2D COSY spectra [52]. All the MRN protons displayed chemical shifts between 6.25 and 7.55 ppm, which are free of the broad and unsolved peaks of CDs. Therefore, the formation of the MRN/CDs inclusion complexes was deduced from the chemical shift changes observed in ^1^H NMR of the MRN aromatic protons previously measured in the free state. Figure 5 shows the stacked portions of the ^1^H NMR spectra of MRN, together with the HP-*β*-CD and SBE-*β*-CD inclusion complexes. In Table 1, we reported the chemical shift of free and complexed MRN in a 1:1 molar ratio with both CDs.

**Figure 6:**
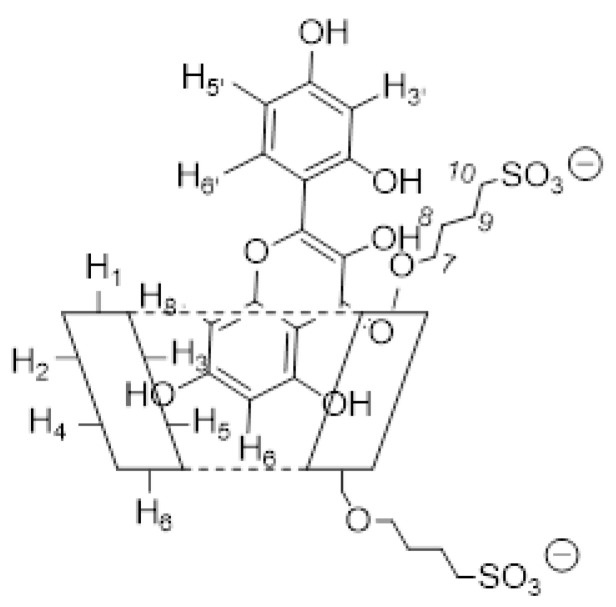
Proposed geometry of the MRN/SBE-*β*-CD inclusion complex as defined by ^1^H NMR analysis.

As regards the MRN/HP-*β*-CD complex, the NMR data obtained by us (performed in a 1:1 D2O/MeOD solution) are in agreement with those reported in the literature (D2O) [43], according to which the ring A of MRN is included into the hydrophobic HP-*β*-CD cavity form the wide side, with the H6 proton facing the CD narrow side, whilst the B ring protrudes towards the primary hydroxypropyl group. Also, the inclusion of MRN in the SBE-*β*-CD cavity was confirmed by changes in the chemical shifts of the guest protons in comparison with the chemical shift of the same protons in the free molecules. A downfield of the aromatic H6 and, especially, H8 MRN protons (Δδ = 0.11) indicated that they were close to an electronegative atom, like oxygen, and therefore, MRN penetrates into the cavity of the SBE-*β*-CD with the A–C rings. Furthermore, H3ʹ, H5ʹ, and H6ʹ protons undergo a significant positive chemical shift (Table 1), indicating that the B ring probably is in close contact with the sulfobutylether groups (Figure 6). The higher-than-usual chemical shift changes observed for the MRN/SBE-*β*-CD inclusion complex are in agreement with those previously reported by us [52].

#### Dynamic Light Scattering (DLS) and Zeta Potential (ζ)

As demonstrated by different authors [53,54,55,56], CDs are able to self-assemble in aqueous solution, forming nanoaggregates of sizes ranging from 100 to 300 nm [57,58]. These superstructures increase in size by increasing the CD monomer concentration. Due to the large interest that CD-based nanoaggregates have in the pharmaceutical field [59] and their ability to positively affect the permeation of the drug through the viable membrane [60], the sizes and ζ values of HP-*β*-CD- and SBE-*β*-CD-forming nanoaggregates were investigated.

As reported in Table 2, we obtained nanoaggregates in the range of 160 to 300 nm, as early as the lowest concentration assayed (0.01 mg/mL) [57]. It is possible to notice that the size of the nanoaggregates is significantly related to the different substituent moieties on the structure of the native *β*-CD as well as the presence of the guest. In particular, free SBE-*β*-CD, at concentrations below 1 mg/mL, forms nanoaggregates with lower sizes with respect to free HP-*β*-CD at the same concentrations. This effect could be attributed to the negative charge of the sulfobutyl group, which could induce the formation of smaller nanoaggregates due to the mutual repulsion of the monomers [54]. Furthermore, MRN exerts a different influence on the self-assembly mechanism of the two modified CDs.

Probably, the interaction of MRN with sulfobutyl groups produces a reduction in the mutual repulsion between SBE-*β*-CD monomers, with a consequent increase in the nanoaggregate sizes. In this case, nanoaggregates are larger than the ones observed for the MRN/HP-*β*-CD complex. For this latter system, nanoaggregate sizes decrease by increasing the concentration in the solution of the complex. It is conceivable that the interaction involved between MRN and the macrocycle reduces the extension of interactions that can occur between the HP-*β*-CD monomers, with a consequent reduction in the nanoparticles’ sizes.

Moreover, all CD-based nanoaggregates showed negative *ζ* values (Table 2). This could be related to the free hydroxyl groups of the CDs and MRN [61]. The absolute *ζ* values are, for all investigated formulations, greater than 20. This result could be considered the minimum value to keep single particles separated due to electrostatic repulsion [62]. Thus, the dispersed particles could be considered physically stable.

### 3.2. Solid-State Characterization

#### 3.2.1. Wide-Angle X-ray Diffraction (WAXD)

**Figure 7:**
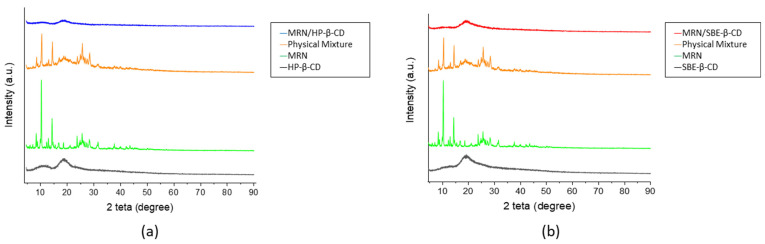
WAXD patterns of (**a**) HP-*β*-CD (black line), free MRN (green line), physical mixture (PM, orange line), and MRN/HP-*β*-CD inclusion complex (blue line); (**b**) SBE-*β*-CD (black line), free MRN (green line), physical mixture (PM, orange line), and MRN/SBE-*β*-CD inclusion complex (red line).

The formation of an inclusion complex between CDs and a crystalline guest was investigated by means of WAXD analysis. The loss of crystallinity of the guest molecule is an indicator of the formation of the complex [63]. MRN, HP-*β*-CD, SBE-*β*-CD, their physical mixtures, and inclusion complexes WAXD patterns are in Figure 7.

As reported in Figure 7, the MRN profile is characterized by sharp and intense flexes, indicating the highly crystalline nature of the organic compound. On the other hand, the WAXD patterns of HP-*β*-CD and SBE-*β*-CD show both an amorphous nature with two broad flexes [64]. The physical mixture patterns (orange line in Figure 7a and in Figure 7b) could be defined as a superposition of MRN and CD patterns (green line and black line, respectively, in Figure 7a and in Figure 7b), being easily assignable to both the amorphous CDs and the crystalline MRN. Patterns related to the inclusion complexes reported in Figure 7a,b reveal that all crystalline features of MRN had vanished. These findings seem to suggest that MRN was entrapped in the cavity of HP-*β*-CD and SBE-*β*-CD.

#### 3.2.2. Thermogravimetric Analysis (TGA)

**Figure 8:**
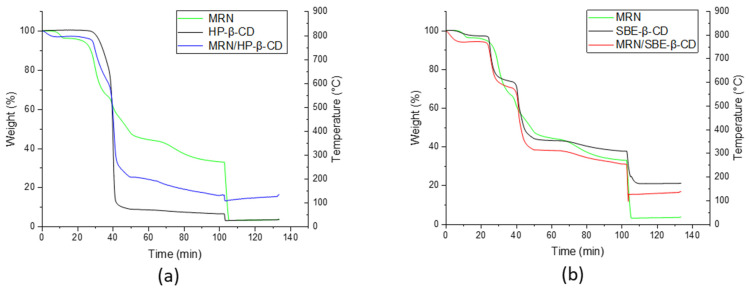
TGA curves of (**a**) HP-*β*-CD (black line), free MRN (green line), and MRN/HP-*β*-CD inclusion complex (blu line); (**b**) SBE-*β*-CD (black line), free MRN (green line), and MRN/SBE-*β*-CD inclusion complex (red line).

The thermal properties of MRN, HP-*β*-CD, SBE-*β*-CD, and their inclusion complexes were also investigated by thermogravimetric analysis. TGA curves are shown in Figure 8. Data from TGA are reported in Table 3.

The decomposition profile for all the samples reveals two main steps in the following temperature ranges: (i) the mass loss under 150 °C can be attributed to the water loss associated with the CDs; (ii) the second process, in the range of 150–550 °C can be related to the degradation of HP-*β*-CD and SBE-*β*-CD or pure MRN. In addition, the inclusion complexes underwent weight losses in three stages: (i) dehydration of water molecules, (ii) decomposition of HP-*β*-CD and SBE-*β*-CD, and (iii) probably due to the decomposition of MRN. It is worth mentioning that a slight modification of the degradation temperature of CDs to inclusion complexes is evidenced. This last finding would suggest the formation of inclusion complexes. As can be seen, the thermal stability of MRN is increased when this is complexed with the CDs.

#### 3.2.3. FT-IR Analysis

FT-IR is one of the most useful ways to identify the formation of inclusion complexes. Indeed, the functional groups of molecules show typical FT-IR absorption bands, and variations in these characteristic bands of the guest molecule (peak intensity, changes in wavenumbers, disappearance, magnification) suggest the formation of inclusion complexes [65]. In this work, FT-IR spectra were registered at different temperatures in order to determine the stability of the complexes and to support their formation.

**Figure 9:**
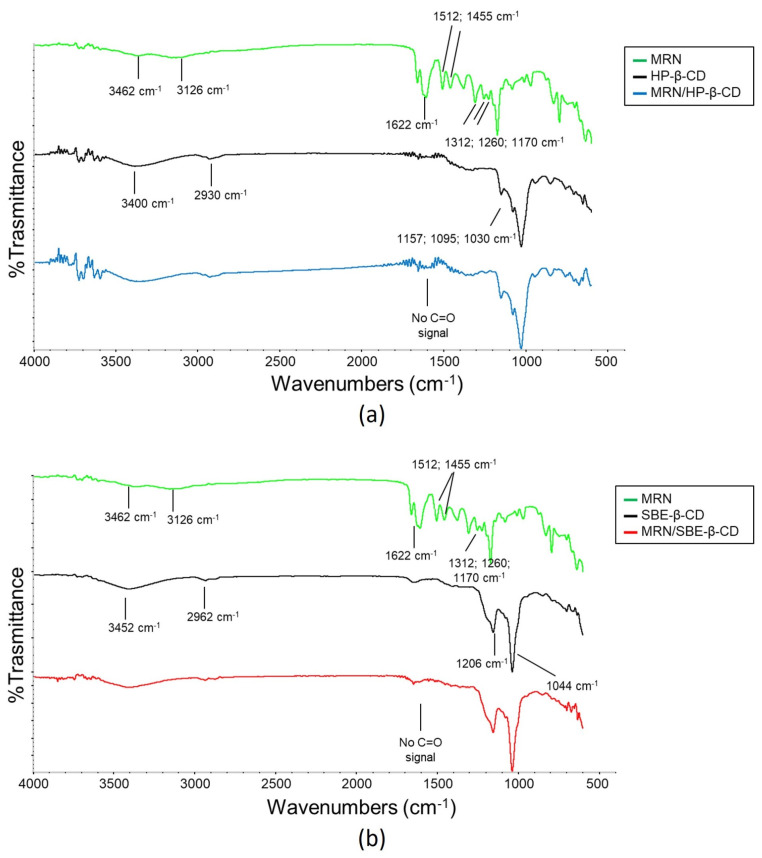
FT-IR spectra recorded at 25 °C of (**a**) free MRN (green line), HP-*β*-CD (black line), and MRN/HP-*β*-CD inclusion complex (blue line); (**b**) free MRN (green line), SBE-*β*-CD (black line), and MRN/SBE-*β*-CD inclusion complex (red line).

The FT-IR spectra recorded at 25 °C of MRN, HP-*β*-CD, SBE-*β*-CD, and their inclusion complexes are shown in Figure 9.

As reported in Figure 9, the FT-IR spectrum of MRN consists of the prominent absorption bands related to the hydroxyl groups at 3373 cm^−1^ and 3145 cm^−1^. The band due to the carbonyl group (C=O stretching vibration) is at 1626 cm^−1^. The C=C stretching vibrations typical of aromatics rings are at 1507 cm^−1^ and 1459 cm^−1^. The C–O–C stretching vibrations (ether group) are at 1308 cm^−1^, 1255 cm^−1^, and 1173 cm^−1^.

The spectrum of HP-*β*-CD is characterized by the presence of (i) the bands due to the hydroxyl groups stretching at 3370 cm^−1^ and for the H–O bending vibration at 1660 cm^−1^; (ii) –CH stretching vibration and –CH_2_ asymmetrical stretching vibration are at 2920 cm^−1^; and (iii) at 1153, 1079, and 1029 cm^−1^ are the bands of C–O stretching.

However, in the MRN/HP-*β*-CD inclusion complex spectrum, some bands of MRN disappeared; for example, the small characteristic absorption bands of the MRN of 400–1055 cm^−1^ disappeared, as well as the bands related to the aromatic groups (1512 and 1455 cm^−1^) and the carbonyl group (1633 cm^−1^). This could be ascribed to the restriction of the vibration of MRN, suggesting that it was entrapped in the cavity of the HP-*β*-CD molecule.

Concerning SBE-*β*-CD, the FT-IR spectrum was characterized by the presence of an absorption band of hydroxyl groups at 3442 cm^−1^ (for –OH stretching vibration); groups CH and CH_2_ attributed to the absorption band at 2940 cm^−1^ (for –CH stretching vibration and –CH_2_ asymmetrical stretching vibration) and another at 1044 cm^−1^ (for C–O stretching vibration). Likewise, in this case, some MRN bands did not appear in the MRN/SBE-*β*-CD inclusion complex spectrum, evidencing the formation of the inclusion complex.

**Figure 10:**
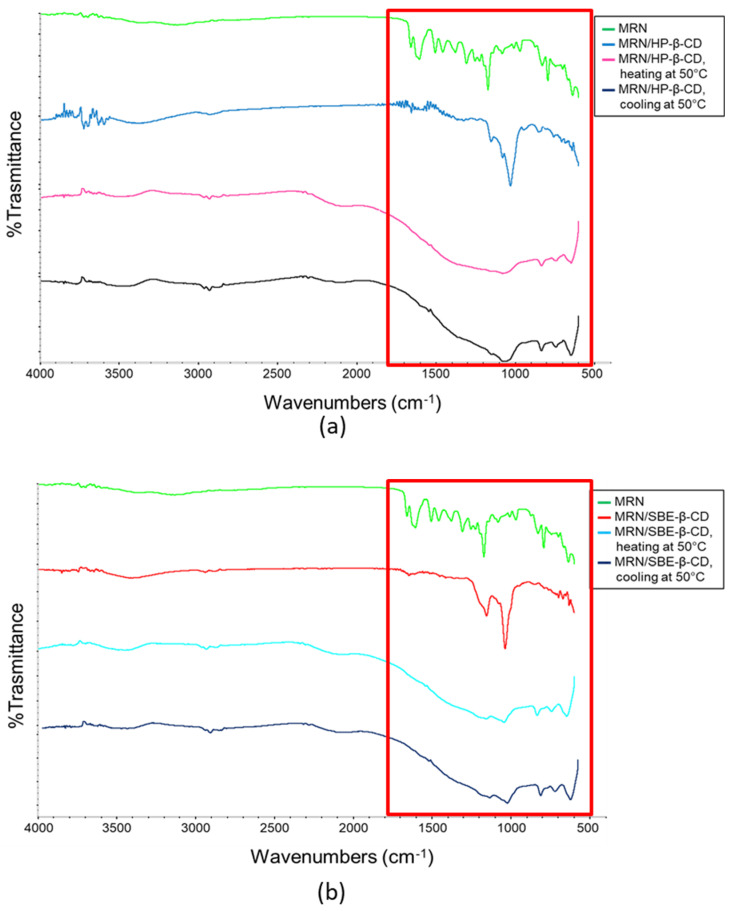
FT-IR spectra recorded at different temperatures of (**a**) free MRN (green line), MRN/HP-*β*-CD inclusion complex at 25 °C (blue line), MRN/HP-*β*-CD inclusion complex heated up to 50 °C (pink line), and MRN/HP-*β*-CD inclusion complex at 50 °C, after the heating treatment up to 100 °C (black line); (**b**) free MRN (green line), MRN/SBE-*β*-CD inclusion complex at 25 °C (red line), MRN/SBE-*β*-CD inclusion complex heated up to 50 °C (light blue line), and MRN/SBE-*β*-CD inclusion complex at 50 °C, after the heating treatment up to 100 °C (dark blue line).

As reported previously, a scanning temperature analysis was conducted. In particular, FT-IR spectra were collected at different temperatures, heating the samples with a rate of 10 °C/min up to 100 °C. The comparison among free MRN and the inclusion complexes with HP-*β*-CD and SBE-*β*-CD are reported in Figure 10.

In Figure 10, it is possible to notice that when the spectra are collected at room temperature, the complex’s features are clearly visible. During the temperature scanning analysis, it is possible to observe the formation of a strongly widened band which is typical of cellulosic compounds. The spectra were registered up to 100 °C.

Once the temperature was reached, the analyzed mixture was slowly cooled down to room temperature. The spectra collected during the cooling phase tend to become increasingly similar to the spectra of the complexes registered before the heating phase. This evidence allows us to highlight the formation of the inclusion complexes and, at the same time, allows us to evaluate the thermal stability of the complexes as a function of the temperature.

### 3.3. Biological In Vivo Studies

#### 3.3.1. Antiangiogenic Activity on Chick Chorioallantoic Membrane (CAM)

As reported by Jung et al. [26], MRN shows high antiangiogenic activity in the chick chorioallantoic membrane (CAM) test. The CAM test is a suitable method to study the angiogenic process and, consequently, the angiogenesis-dependent processes, such as tumor growth [66]. Thus, it can be efficaciously used to evaluate the anticancer activity of a drug in terms of inhibition of new vessel development [67].

**Figure 11:**
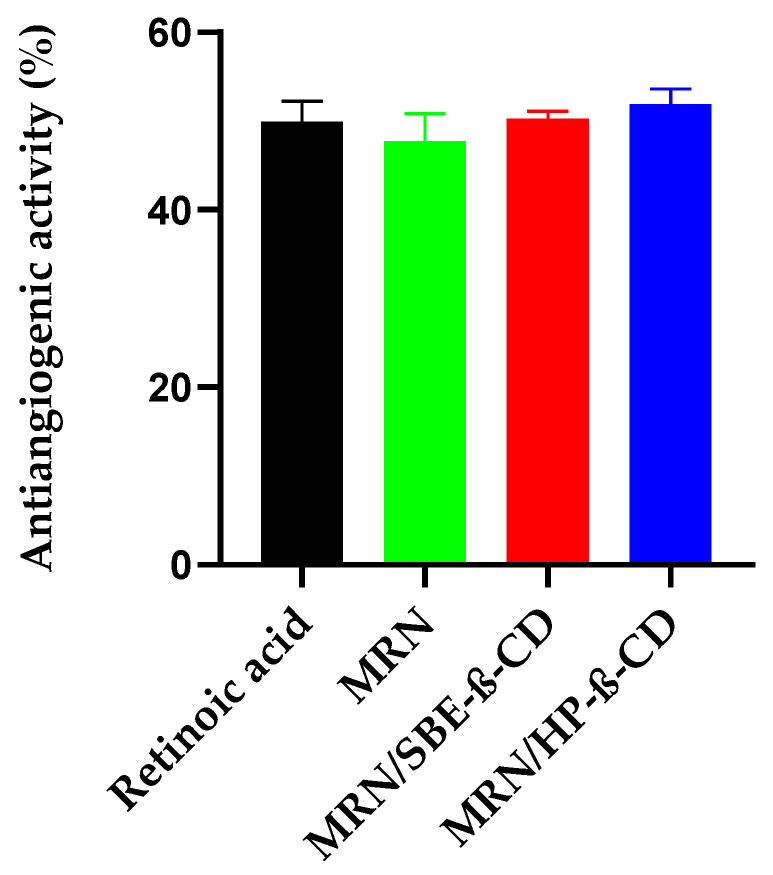
The antiangiogenic activity of free and complexed MRN as compared to retinoic acid (positive control) was calculated as inhibition percentage versus negative control in a set of experiments (n = 6) ± standard deviation (SD).

**Figure 12:**
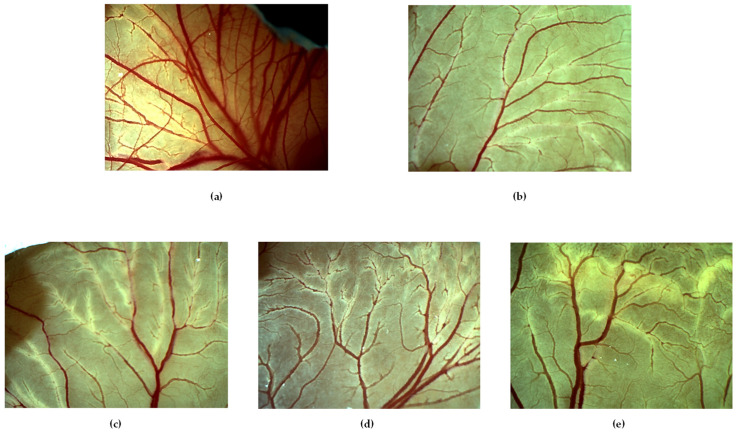
Representative photomicrographs of the chick embryo CAM treated with free and complexed MRN at 33 μM. Samples (100 μL/egg) were applied directly on CAM surface. Retinoic acid was used as positive control (3 μM). (**a**) Negative control; (**b**) retinoic acid; (**c**) free MRN; (**d**) MRN/SBE-*β*-CD inclusion complex; (**e**) MRN/HP-*β*-CD inclusion complex.

We used the CAM test to evaluate the influence of HP-*β*-CD and SBE-*β*-CD on the antiangiogenic activity of MRN. Based on the results obtained by other authors, we assayed free and complexed MRN at 1 μg/egg (33 μM) [26]. The obtained results, as inhibition percentage of angiogenesis with respect to the control, were reported in Figure 11, whilst representative microscopic images of the CAM vascular network are presented in Figure 12.

Retinoic acid was used as a positive control for its well-known antiangiogenic activity (3 μM, about 50% of inhibition). MRN showed antiangiogenic activity like the positive control, showing a reduction in vessel number and bifurcations with respect to the non-treated eggs (negative control) (Figure 10) and a percentage of inhibition of about 48% (Figure 11). As expected, no influence was exerted by the two CDs on angiogenesis, showing a vessel’s growth like untreated eggs. Concerning the inclusion complexes, they apparently did not show advantages with respect to the administration of free MRN due to the comparable antiangiogenic activity observed (Figure 11 and Figure 12). However, some considerations must be made. Firstly, only the drug in solution is active; secondly, only the non-complexed drug can cross the viable membranes and show its antiangiogenic activity. In our study, free MRN is dissolved in DMSO and after diluted in Tris buffer (pH 7.4); thus, all the assayed dose is in solution and totally available for absorption through the chorioallantoic membrane. Concerning MRN administered as inclusion complexes, it is freely soluble in Tris buffer (pH 7.4), but in this case, the dose of MRN available for absorption is that released by the complexes and depends on their *Kc* value. As observed by our characterization studies, both inclusion complexes showed high *Kc*, with a consequent limitation of the amount of free MRN released. In this way, the comparable antiangiogenic activity observed for the inclusion complexes with free MRN must be considered a positive result since free MRN is an insoluble drug with a very slow dissolution rate (see Figure 2). This drawback limits the freedom to formulate MRN in various dosage forms, forcing formulations as tablets or suspensions and producing erratic and incomplete bioavailability. On the contrary, based on the MRN/CDs inclusion complexes, an aqueous liquid formulation can be obtained with potentially high effectiveness.

#### 3.3.2. Zebrafish Embryos Model

Recently, zebrafish embryos have received great attention as an emerging model to predict toxic activity [68] and to assay anti-oxidant [69] or antiangiogenic activity [70] of substances. Zebrafish grow quickly and are transparent, permitting a direct vision of blood flow and vascular development. This latter can be easily monitored by quantification of endogenous alkaline phosphatase activity, released by the endothelial cells, and representing a marker of vessel growth [71].

The antiangiogenic effect of free MRN and the inclusion complexes were compared with that of 2-methoxyestradiol (ME), an endogenous metabolite of 17*β*-estradiol having known antiangiogenic and antitumor properties [72], which determined an inhibition of 49.50% at 20 µM in our experimental conditions. Firstly, we determined the tolerated MRN dose by treating zebrafish embryos with increasing concentrations of free and complexed MRN, ranging from 50 to 150 μM. Free MRN produced no toxic effect until the concentration of 75 μM, showing high vitality of the embryos, whilst the higher assayed concentrations produced high mortality of the subjects of about 80% and 95% for the doses of 100 and 150 μM, respectively. The treatment of zebrafish embryos with the inclusion complexes did not produce a toxic effect at all assayed concentrations, and at the end of the experiment, all embryos were viable, demonstrating a protective effect produced by the CDs.

**Figure 13:**
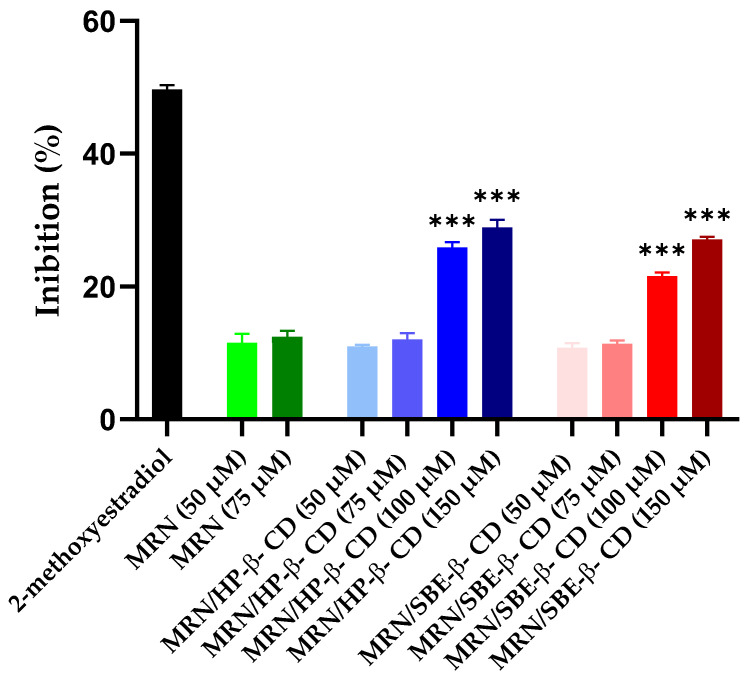
The antiangiogenic activity of free MRN at a dose of 50 and 75 μM, while the complexed drug was assayed at 50, 75, 100, and 150 μM, as compared to 2-methoxyestradiol (positive control). The antiangiogenic activity was expressed in terms of reduced activity of endogenous alkaline phosphatase. Results are expressed as mean values of six different experiments from six different batches ± standard deviation (SD). MRN/SBE-*β*-CD and MRN/HP-*β*-CD at 100 and 150 µM are statistically significant with respect to free MRN at the tested doses (*** *p* < 0.001).

Based on tolerability studies, we assayed the antiangiogenic activity of free MRN at a dose of 50 and 75 μM, while the complexed drug was assayed at 50, 75, 100, and 150 μM doses. The antiangiogenic activity was expressed in terms of reduced activity of endogenous alkaline phosphatase, and the obtained results are presented in Figure 13.

Free MRN showed a modest antiangiogenic activity, non-dose-dependent, with about 15% inhibition. Similar activity was obtained for the inclusion complexes at the same doses. As explained for CAM studies, the complexation reduces the amount of free MRN available to explicate the pharmacological activity; thus, similar or lesser activity with respect to free MRN was expected. A different trend was observed at higher concentrations. MRN complexed with both CDs showed an inhibitory dose-dependent effect on vessel growth in the embryos treated with doses of 100 and 150 µM of MRN, evidencing an important effect exerted by the macrocycles and due to the increase in MRN water solubility and probably to the ability of macrocycles to interact with the viable membrane, improving the permeation of solubilized drug.

## 4. Conclusions

In this work, we realized the inclusion complexes of MRN with HP-*β*-CD and SBE-*β*-CD with the aim of improving the water solubility and antiangiogenic activity of the natural drug MRN. The complexes were investigated both in solution and in the solid state, showing comparable affinity of MRN towards the two different CDs. Stable inclusion complexes were obtained that positively affect the water solubility of the drug, permitting the design of liquid formulations for oral or parenteral administration. CAM and zebrafish embryo models demonstrated that CDs are able to improve the antiangiogenic activity of MRN in vivo. Even if other studies are needed, the results obtained in this study show the high potentiality of the MRN/CD inclusion complexes for the treatment of angiogenesis-dependent diseases such as cancer.

## Figures and Tables

**Table 1:** ^1^H NMR chemical shifts in δ and Δδ of MRN protons in free state and MRN/SBE-*β*-CD and MRN/HP-*β*-CD 1:1 complexes [8 mM in D_2_O/MeOD (0.5/0.5 mL) solution]; for doublet, double–doublet, or multiplet, the reported δ refer to the centered signal.

Protons	MRN	MNR/SBE-*β*-CD	Δδ *	MRN/HP-*β*-CD	Δδ *
H-6	6.316 (d)	6.338	0.022	6.311	−0.005
H-8	6.466 (d)	6.625	0.159	6.490	0.024
H-3′	6.535 (d)	6.575	0.040	6.532	−0.003
H-5′	6.601 (dd)	6.624	0.023	6.600	−0.001
H-6′	7.482 (dd)	7.501	0.019	7.490	0.008

* Δδ = δ_complex_ − δ_free_.

**Table 2:** Sizes and ζ values of nanoaggregates based on HP-*β*-CD, SBE-*β*-CD, and their inclusion complexes with MRN. The inclusion complexes were in a 1:3 molar ratio. The concentration of the inclusion complexes refers to the CD.

Sample	Size (nm)	PDI	ζ (mV)
HP-*β*-CD 1 mg/mL	239.0 ± 33.76	0.35	−31.3 ± 2.3
HP-*β*-CD 0.1 mg/mL	221.2 ± 36.92	0.48	−19.3 ± 2.4
HP-*β*-CD 0.01 mg/mL	193.9 ± 20.27	0.59	2.3
MRN/HP-*β*-CD 1 mg/mL	103.2 ± 11.00	0.51	−23.9 ± 0.85
MRN/HP-*β*-CD 0.1 mg/mL	130.1 ± 69.32	0.77	−19.2 ± 3.61
MRN/HP-*β*-CD 0.01 mg/mL	187.1 ± 26.62	0.59	−16.3 ± 1.55
SBE-*β*-CD 1 mg/mL	247 ± 25.07	0.63	−19.1 ± 2.5
SBE-*β*-CD 0.1 mg/mL	123.8 ± 4.987	0.88	n.d.
SBE-*β*-CD 0.01 mg/mL	166.9 ± 13.28	0.77	n.d.
MRN/SBE-*β*-CD 1 mg/mL	305.4 ± 83.12	0.41	−28.4 ± 3.5
MRN/SBE-*β*-CD 0.1 mg/mL	195.3 ± 27.55	0.43	−26.9 ± 2.25
MRN/SBE-*β*-CD 0.01 mg/mL	255.2 ± 43.28	0.53	−26.1 ± 4.9

**Table 3:** Mass losses (mass %) of free MRN, HP-*β*-CD, SBE-*β*-CD, and their inclusion complexes, from TGA analysis.

Sample	Mass Loss %				Residue
	T < 150 °C	150 °C < T < 550 °C	550 °C < T < 900 °C	T > 900 °C	
MRN	3.50	48.0	15.4	29.4	3.70
HP-*β*-CD	0.50	91.0	2.40	2.90	3.20
MRN/HP-*β*-CD	2.80	71.7	9.20	2.96	13.41
SBE-*β*-CD	2.10	53.3	6.90	16.7	21.0
MRN/SBE-*β*-CD	5.84	61.2	7.60	19.2	6.16

## Data Availability

Data are contained within the article.
